# Feasibility, Acceptability, and Impact of Recovery-Oriented Practices in an Italian Community Mental Health Service: A Pilot Study

**DOI:** 10.3390/jcm14072280

**Published:** 2025-03-27

**Authors:** Alessandra Martinelli, Tecla Pozzan, Elena Procura, Camilla D’Astore, Doriana Cristofalo, Chiara Bonetto, Mirella Ruggeri

**Affiliations:** 1 Unit of Epidemiological Psychiatry and Digital Mental Health, IRCCS Istituto Centro San Giovanni di Dio Fatebenefratelli, 25125 Brescia, Italy; 2 Section of Psychiatry, Verona Hospital Trust, Azienda Ospedaliera Universitaria Integrata, 37134 Verona, Italymirella.ruggeri@univr.it (M.R.); 3 Mental Health Center, Isola della Scala, Ospedale di Bussolengo, 37063 Verona, Italy; 4 Department of Neurosciences, Biomedicine and Movement Sciences, University of Verona, 37129 Verona, Italychiara.bonetto@univr.it (C.B.)

**Keywords:** recovery, pilot study, mental health recovery star, focus group, community mental health service

## Abstract

**Background:** Over the past decade, Italy has made progress in adopting recovery-oriented approaches in mental health care, though full alignment with international guidelines remains incomplete. This study investigates the feasibility, acceptability, and impact of integrating recovery-oriented practices in an Italian Community Mental Health Service (CMHS), focusing on both user and professional perspectives to identify strengths and areas for improvement. **Methods:** A longitudinal pilot study was conducted at the South Verona CMHS. Data on users’ socio-demographic and clinical characteristics, symptoms, functioning, needs, and autonomy were collected at baseline and six-month follow-up. Participants included individuals in supported accommodation and outpatient care. The Mental Health Recovery Star (MHRS) assessed recovery progress. Qualitative data from focus groups and interviews captured users’ and professionals’ experiences. **Results:** Nineteen professionals completed the MHRS with 25 users, who demonstrated significant improvements in MHRS scores (*p* = 0.003), romantic relationships (*p* < 0.001), employment (*p* < 0.001), functioning (*p* = 0.015), psychopathology (*p* = 0.001), functional autonomy (*p* = 0.003), and unmet needs (*p* = 0.026). Qualitative findings emphasized the value of a personalized, holistic approach but noted gaps in follow-up and shared decision-making. Focus groups (30 participants) highlighted recovery as a process of hope, meaning, and empowerment. Participants called for ongoing education, structural changes, and peer-support initiatives. Professionals reported increased motivation. **Conclusions:** Integrating recovery-oriented practices within the South Verona CMHS was both feasible and acceptable. The MHRS positively impacted service users’ personal recovery and professionals’ motivation. The study underscores the need for continued training, structural reforms, and peer-support initiatives to foster lasting changes and enhance CMHS practices.

## 1. Introduction

Since 2005, numerous international recommendations across Europe, such as the Declaration on Mental Health for Europe [1], the European Commission’s Green Paper [2], and the United Nations Convention on the Rights of Persons with Disabilities [3], have underscored the importance of upholding the fundamental freedoms and human rights of individuals with disabilities [4]. The NICE guidelines [5] have emphasized the importance of delivering care to individuals with psychosocial disabilities predominantly within community settings, involving all stakeholders and adopting a flexible and recovery-oriented approach.

Anthony defines recovery as a deeply personal and transformative process that encompasses changes in attitudes, values, feelings, goals, skills, and roles. Despite limitations caused by their psychiatric disorder, individuals can lead a satisfying, hopeful, and meaningful life [6], actively participating in adult roles within their homes, workplaces, educational settings, and other social environments [7,8,9].

The implementation of the recovery model in community mental health services (CMHSs), particularly through evidence-based recovery-oriented practices [10,11,12], has resulted in improved user self-management, self-efficacy, and autonomy [8,13], as well as enhanced health and social outcomes [14,15,16]. These practices are considered essential for adapting CMHS to the needs of the current century and correlate positively with treatment outcomes improvement and reduced health costs [10,17,18,19].

Current CMHS objectives should aim to provide continuous care for individuals with mental disorders, focusing on personal recovery and rehabilitation [20], personalized and shared treatments [17,21,22,23] that integrate the recovery paradigm with evidence-based medicine [7,24], and valorize the user’s and their family’s experiences in developing personalized, user-centered projects [5,17,23].

In Italy, there is potential to foster the adoption of recovery-oriented practices due to the existing tradition of community care [25]. Over the past decade, essential elements of the personal recovery approach have emerged [26,27,28,29], including the collection and dissemination of recovery stories to inspire and provide hope to users, families, and professionals [26,30,31,32]. Co-production and peer expertise initiatives have been implemented in various Italian settings [33,34,35], while recovery-oriented interventions have been integrated [36] into critical life dimensions such as work [37,38,39]. Moreover, specific training programs on personal recovery have been developed for mental health professionals [25], and tools for facilitating and assessing personal recovery have been created or translated from other languages [40,41,42,43,44].

However, the implementation of recovery-oriented practices in Italian CMHSs has not fully met international recommendations. The adoption of the recovery paradigm remains a challenge for many professionals who are accustomed to paternalistic approaches [26,45]. Treatment and intervention specificity is often lacking, and the establishment of care pathways that prioritize user real needs and satisfaction can be difficult [25,31,46].

Jim van Os and colleagues have suggested the need for pilot projects to investigate the feasibility and integration of recovery-oriented practices in clinical work [21,47].

The objective of this paper is to present a small-scale study that explores the feasibility, acceptability, and impact of implementing and integrating recovery-oriented practices within an Italian CMHS. The present pilot study employed the Mental Health Recovery Star (MHRS) within a longitudinal framework and focus groups to guide routine practices and interventions toward a personalized, recovery-oriented approach.

## 2. Materials and Methods

### 2.1. Study Design

All the study procedures comply with the ethical standards of the relevant national and institutional committees on human experimentation and with the Helsinki Declaration of 1975 (version 2008): procedures involving patients were approved by the Research Ethics Committee of the University Hospital Trust of Verona (reference 34950, 30 May 2018). Written informed consent was obtained from all patients and mental health professionals.

The current pilot study employed the MHRS in a longitudinal design alongside two focus groups to guide everyday practices and activities toward a personal recovery-oriented approach. The pilot study was conducted from May 2017 to October 2018 at the South Verona CMHS in Italy, which follows a long tradition of evidence-based medicine and the bio-psycho-social model [48]. Following deinstitutionalization, CMHSs became essential in Italy’s mental health system, providing diagnosis, care, and support. Each district-based CMHS serves around 100,000 people to enhance accessibility [49]. Across Europe, coverage ranges from 80,000 people in high-income countries to over 2 million in low-income countries [48,50]

### 2.2. Longitudinal Study

This prospective cohort study took place from October 2017 to October 2018, with data gathered at the time of recruitment and again after six-month follow-up, following standard evaluation practices in Italian rehabilitation settings.

#### 2.2.1. The MHRS

The MHRS, developed by Triangle Consulting in 2007 for the Mental Health Providers Forum [51], is a tool designed to support personal recovery by fostering “expert-to-expert” partnerships between users and professionals. It facilitates shared decision-making (SDM) and helps set and track recovery goals [51,52,53]. Adopted in the UK [54] and internationally, it was translated into Italian in 2013 with over 8000 professionals trained in its use [40,55].

The MHRS employs a 10-point star-shaped schema, each point representing a key life domain: Managing mental health, Self-care, Addictive behavior, Living skills, Work, Responsibilities, Identity and self-esteem, Trust and hope, Social networks, and Relationships. Users and their key professionals assess progress using the ‘Scale of Change’, based on Prochaska and DiClemente’s Transtheoretical Model [56], which outlines five recovery stages:

- Stuck (phases 1–2): feeling unable to cope or unwilling to seek help.
- Accepting help (phases 3–4): acknowledging the problem and seeking support.
- Believing (phases 5–6): gaining confidence in change and taking action.
- Learning (phases 7–8): experimenting with new approaches.
- Self-reliance (phases 9–10): managing goals independently.

Following completion, users and professionals collaborate on a care plan focusing on up to three goals. Despite concerns about inter-rater reliability, the MHRS demonstrates strong internal consistency and validity. Its true strength lies in its recovery-oriented, collaborative approach, which enhances therapeutic relationships and personal empowerment in mental healthcare [40,54,57,58,59,60,61].

#### 2.2.2. Participants

Eligible participants were mental health professionals from South Verona CMHS who were trained in the MHRS, willing to complete assessments at two time points, and able to recruit at least one service user under their care. Of the 45 professionals trained in MHRS between May and October 2017, 19 were recruited. The remaining 26 were excluded due to not working at South Verona CMHS (15), declining participation (8), or being unable to recruit a service user (3). The majority were female (78.9%), over a third were psychiatry residents (36.8%), and most (73.6%) worked in multidisciplinary community teams. Their average tenure was 137 months (SD 122.2).

Throughout the study, professionals received monthly education and supervision from certified MHRS trainers to ensure consistent use of the tool. Twelve meetings, attended by 2 to 23 participants, covered data collection, recovery principles, MHRS implementation, and training in motivational interviewing and shared decision-making. If needed, trainers assisted professionals in using the MHRS with service users.

Service users were eligible if they had a trained key professional at South Verona CMHS, lived within the service catchment area, were aged 18–65, and consented to assessments at two time points. Exclusion criteria included moderate/severe intellectual disability [62] or psychiatric hospitalization during recruitment (severity assessed by the key professional, using assessments such as the HONOS with multiple items rated as severe or very severe). Key professionals identified and obtained consent from eligible participants, resulting in the recruitment of 25 service users [62].

#### 2.2.3. Assessments

Socio-demographic, service use, and clinical data of service users were obtained from the Verona Department of Mental Health (DMH) database and the South-Verona Psychiatric Case Register [63]. The assessment tools were selected by the research team in collaboration with certified MHRS trainers and experienced rehabilitation staff, ensuring alignment with standard evaluation tools commonly used in South Verona CMHS rehabilitation settings. Following specialized training and under research team supervision, key professionals completed standardized assessments at baseline (BL) and follow-up (FU), including the following:

- Personal and Social Functioning Scale (FPS) [41,42]: Evaluates personal and social functioning across four domains—socially useful activities, personal/social relationships, self-care, and aggressive behavior. Scores range from 1 to 100, with higher values indicating better functioning.
- Health of the Nation Outcome Scale (HoNOS) [64]: Assesses psychopathology and social functioning across 12 items covering behavioral, daily living, mental health, and socio-occupational difficulties. Scores range from 0 (no problem) to 4 (very severe), with higher totals reflecting greater severity.
- Monitoring of the Path of Rehabilitation (MPR) staff version [65,66]: Measures functional autonomy in daily activities such as self-care, housework, shopping, and social engagement. Scores range from 0 to 12, with higher scores indicating greater independence.

Both service users and key professionals, with research assistance, completed the following tools.

**Figure 1:**
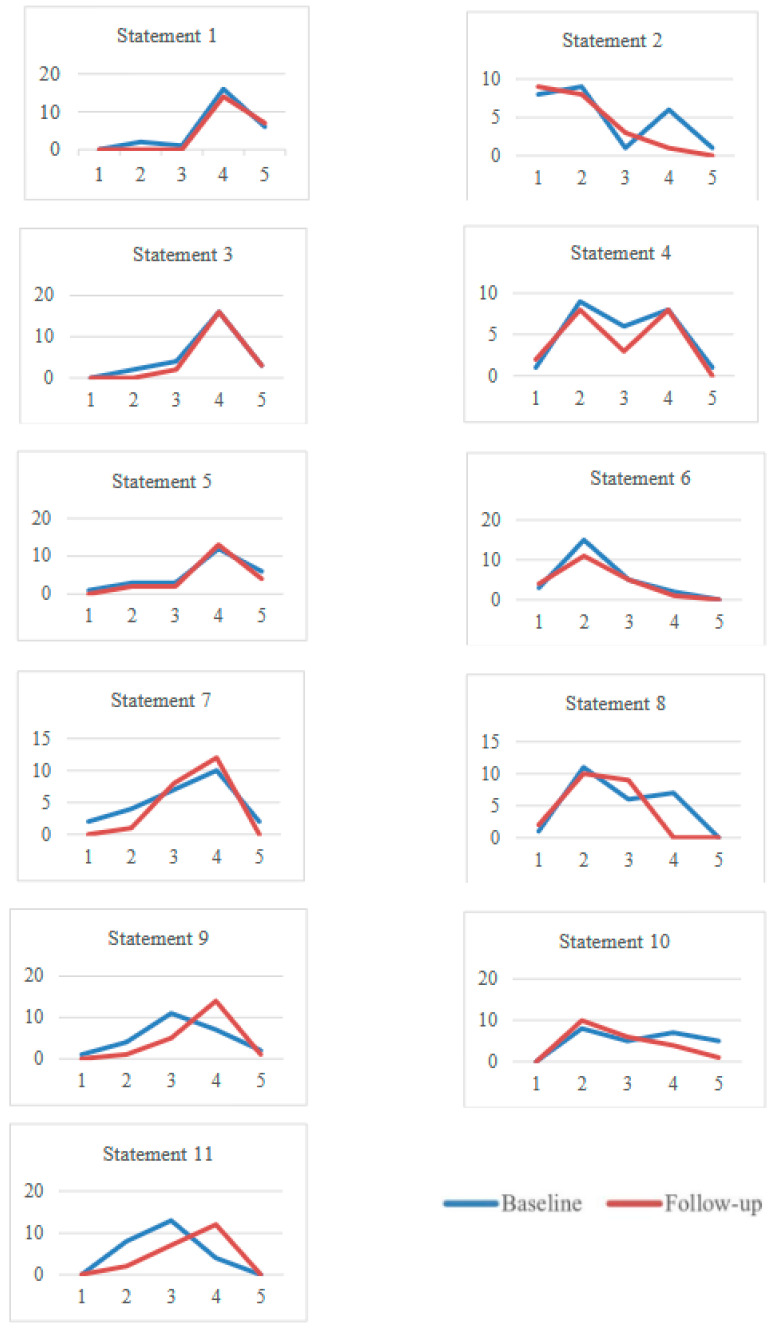
Key professionals’ impact assessment about the recovery implementation in the CMHS at BL and FU. Statements 1, 3, 5, 7, 9, and 11 were positive, while statements 2, 4, 6, 8, and 10 were negative. Likert scale, 1 = Strongly Disagree, 2 = Disagree, 3 = Neither agree nor disagree, 4 = Agree, 5 = Strongly Agree. Staff statements on impact assessments were the following. 1. I feel that service users can clearly communicate their support needs to me; 2. I’m not as motivated as I used to be to encourage positive service user engagement; 3. I feel that I am supporting service users on things that really matter to them; 4. I don’t know how much the service users I work with have progressed since they joined the service; 5. Supervision is used to help me think through my key work and support planning; 6. I often feel that I don’t know how to communicate with service users about the things that matter to them; 7. I can clearly see and evidence how the support needs of service users have changed in the time I have worked with them; 8. I don’t know how the service users I work with feel about the support I provide; 9. I feel service users are actively leading in their own recovery; 10. The impact the work I do has on service users’ lives is not picked up by current monitoring methods; 11. Our service reflects the needs and aspirations of service users and it is enriched by their contributions.

**Figure 2:**
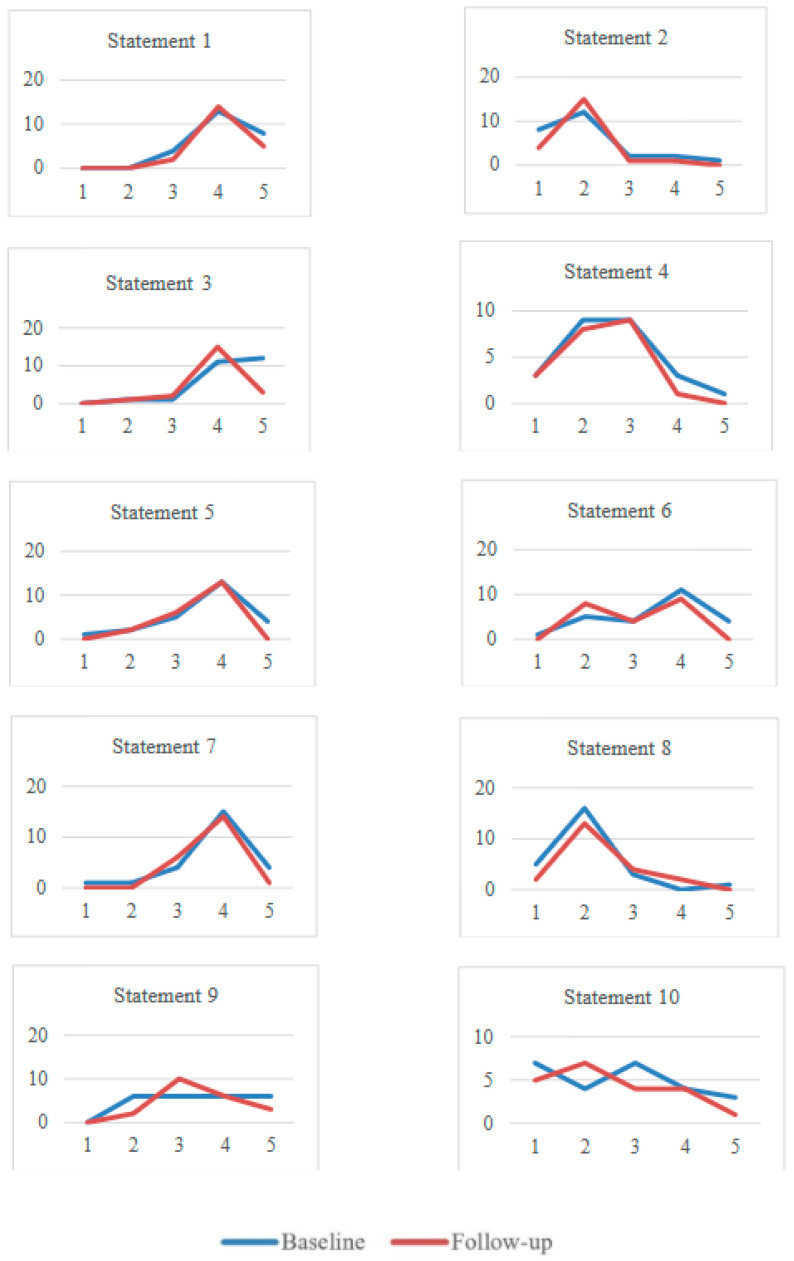
Service users’ impact assessment about the recovery implementation in the CMHS at BL and FU. Statements 1, 3, 5, 7, and 9 were positive statements about the service, whereas statements 2, 4, 6, 8, and 10 were negative. Likert scale, 1 = Strongly Disagree, 2 = Disagree, 3 = Neither agree nor disagree, 4 = Agree, 5 = Strongly Agree. Service users’ statements of impact assessments were the following. 1. I feel listened to when I talk about what support I need; 2. Positive engagement is difficult for me as staff aren’t interested in what I want to do; 3. Staff make me feel relaxed and welcome and I feel confident to approach them for support; 4. I don’t know how much I’ve progressed since joining the service; 5. I am made to feel important and that my opinions matter; 6. There are important areas in my life that I don’t know how to bring up in key working sessions; 7. I feel satisfied with the advice and support I receive relating to education, training and employment; 8. There aren’t enough opportunities to feedback on the support I receive; 9. I feel in control of my life, the decisions I make and the support I get; 10. I feel that I am stuck and don’t know what my next step should be.

- Camberwell Assessment of Need (CAN) staff and patient version [67,68]: Assesses 22 items in four domains (health, basic needs, services, and functioning), categorizing needs as met (score 1) or unmet (score 2), with an overall ratio of met to unmet needs.
- Impact assessment [69]: Evaluates the effects of recovery implementation in CMHS through positive and negative statements rated on a 5-point Likert scale. Staff completed 11 statements, while service users responded to 10 (Figure 1 and Figure 2).
- An acceptability and feasibility assessment: Conducted at the study’s conclusion to assess the usability of MHRS in clinical practice. Participants rated the difficulty of using MHRS and its effectiveness in a recovery-oriented approach on a 5-point Likert scale. Staff completed 18 statements, while service users answered 10 (Table 1).

#### 2.2.4. Statistical Analysis

Descriptive data were summarized using frequencies, means, and standard deviations. Changes in scores on standardized assessment tools from recruitment to FU were analyzed using the paired-samples *t*-test. To assess differences between the first and second evaluation, the *t*-test for repeated measurements was applied. Due to the exploratory nature of this study, no adjustments for multiple comparisons were made.

All tests were two-tailed, with a significance level of 0.05. Statistical analyses were conducted using SPSS version 25.

### 2.3. Recovery-Oriented Focus Groups at the South Verona CMHS

Two recovery-oriented focus groups were conducted to explore the feasibility, acceptability, and impact of recovery-oriented practices. This qualitative approach explored reactions to their implementation in Verona DMH [70] while promoting an “expert-to-expert partnership” and SDM between users and professionals [23,71].

Facilitated by two psychologists and a psychiatry resident, each one-hour session included pre-planned questions, brainstorming, and case studies, with discussions evolving based on emerging themes. Tools like a projector, whiteboard, and audio recording (with consent) supported the process. Participants, including mental health professionals, trainers, and service users, were recruited through an open call.

- First focus group (June–September 2017, six meetings, avg. 12 participants): Focused on recovery education and MHRS training for recent trainees at South Verona CMHS.
- Second focus group (January–May 2018, eight meetings, avg. 17 participants): Explored strategies for implementing the recovery paradigm and forming a collaborative group within Verona DMH.

Audio recordings, facilitator notes, and session summaries were analyzed using qualitative thematic methods, including transcription, coding, and triangulation for reliability.

## 3. Results

### 3.1. Service Users’ Socio-Demographic and Clinical Characteristics from BL to FU

As shown in Table 2, at BL, service users were 41.0 (9.9) y.o., mostly male (60.0%), single (76.0%), unemployed (64.0%), and lived in private accommodations (56.0%). Most had a primary diagnosis of Schizophrenia Spectrum Disorder (68.0%) with a mean of 16 years (CI 12.5–19.5) of contact with CMHS, an intake of 2.9 (SD 1.6) psychotropic drugs, and a mean of 0.3 (0.5) admissions in the acute ward in the previous six months. About a quarter of users presented a concurrent physical comorbidity (32.0%), and a mean of 0.8 (SD 1.1) had a current addiction.

From BL to FU, a significant number of service users developed a romantic relationship (*p* < 0.001), found a stage/employment/school (*p* < 0.001), and increased addictions (*p* < 0.001). There was an almost significant reduction in acute ward admission (*p* = 0.059) (Table 2).

### 3.2. Recovery Star, Functioning, Psychopathology, Functional Autonomy, and Needs of Care from BL to FU

As shown in Table 3, at BL, most service users, along with their key professionals, negotiated that they were generally in the Believing phase of the Scale of Change (MHRS, 6.1, SD 5.1). The lowest scores were found in Social networks (MHRS, 5.5, SD 2.2), Work (MHRS, 5.5, SD 2.4), and Relationships (MHRS, 5.5, SD 2.6), which were the main focus of the intervention plans (Table 4). The highest score was observed in Responsibilities (MHRS, 8.1, SD 2.3).

During the FU, there was a significant overall improvement in the Scale of Change (MHRS, *p* = 0.003), although service users remained in the Believing phase. Specifically, significant improvements were seen in Physical health and self-care (MHRS, *p* = 0.004), Living skills (MHRS, *p* = 0.012), Work (MHRS, *p* = 0.007), Relationships (MHRS, *p* = 0.018), Responsibilities (MHRS, *p* = 0.050), and Identity and self-esteem (*p* = 0.016) (refer to Table 3).

In the FU, among the 20 (80.0%) couples who defined new goals, there was an increase in goals related to Addictive behavior (Table 4).

From BL to FU, users showed improvements in functioning (FPS, *p* = 0.015), psychopathology (HoNOS, *p* = 0.001), and functional autonomy (MPR, *p* = 0.003), with a greater number of met needs (key-professionals CAN ratio from 2.6 to 3.8; service users CAN ratio from 2.8 to 5.2), along with a significant decrease in unmet needs for users (CAN, *p* = 0.026) (see Table 3).

### 3.3. Assessing the Impact and Feasibility of Implementing MHRS a Recovery-Oriented Approach

Figure 1 displays the impact assessment of professionals from BL to FU. Positive statements increased overall (from 3.6, SD 0.5, to 3.8, SD 0.4), with significant improvements in statement 9 (‘I feel service users are actively leading in their own recovery’; *p* = 0.036) and statement 11 (‘Our service reflects the needs and aspirations of service users and is enriched by their contributions’; *p* = 0.007). Negative statements decreased (from 4.0, SD 0.6 to 3.8, SD 0.5), indicating overall satisfaction with CMHS and an increase in motivation for work with service users.

Users also experienced changes from BL to FU. Positive statements decreased overall (from 4.0, SD 0.6, to 3.8, SD 0.5), with a significant decrease in statement 3 (‘Staff make me feel relaxed and welcome and I feel confident to approach them for support’; *p* = 0.038). Negative statements decreased (from 2.4, SD 0.6) compared to BL (from 2.6, SD 0.7) (Figure 2).

As shown in Table 1, both professionals and users rated MHRS completion as moderately difficult, with adherence to intervention schedules being the most challenging aspect. MHRS facilitated greater user engagement, trust, and problem-solving, with both groups recognizing its role in uncovering hidden resources. While professionals found it beneficial for structuring interventions, some reported difficulties in achieving full collaboration with users.

### 3.4. Focus Groups

The focus groups provided insights into participants’ understanding of recovery-oriented principles and collaborative practices, highlighting their potential to facilitate a more recovery-oriented approach in the South Verona CMHS.

Active participation was observed, with some attendees in both sessions, including current service users, new contacts, and some individuals who returned after dropping out. Two users had to interrupt due to relapses triggered by topics.

In the first focus group, participants demonstrated a clear understanding of recovery concepts and the MHRS. Recovery was conceptualized as a dynamic, personalized process involving hope, meaning, choice, identity, and resilience. The group emphasized the need for tailored support at different stages of change [56] and highlighted empowerment as crucial for self-advocacy and co-creating care pathways. These discussions informed the integration of the MHRS within both therapeutic and rehabilitative contexts, providing a basis for further training and pilot studies aimed at refining recovery-oriented practices.

The second focus group encountered challenges with SDM and the “expert-to-expert partnership” model, which contrasts with traditional professional roles. Despite these challenges, participants engaged with all topics and proposed several improvements, including promoting non-stigmatizing practices, developing self-help groups, and creating training for patients to become peer workers. Structural changes in CMHS were identified as necessary for deeper integration of recovery principles.

Facilitating balanced participation proved difficult at first, with some users voicing grievances and professionals hesitating to engage. However, the use of structured facilitation strategies, including reinforcing participation rules, fostering openness through facilitator self-disclosure, and managing disruptions, helped build trust and led to more productive and constructive exchanges.

### 3.5. Advancements in Recovery-Oriented Practices at South Verona CMHS Post-Pilot Study

After the pilot study, the South Verona CMHS began integrating key elements of the recovery approach, such as referring to service users rather than patients and integrating the MHRS change scale phases into clinical practice.

Despite challenges such as limited recovery education due to the SARS-CoV-2 pandemic and staff turnover, both professionals and service users consistently highlighted the need for more recovery-focused training, particularly with the MHRS tool.

While the formation of a dedicated working group to advance recovery-oriented practices proved difficult, notable progress was made. In early 2017, a self-help group was established, bringing together users and professionals from different Verona CMHSs. This biweekly group expanded over time and provided a platform for discussions on mental health perspectives. The group also supported the development of “The Open Circle”, an unofficial users’ association aimed at advocating for recovery-oriented practices, providing peer support, and combating social exclusion. While not officially registered, the group continues to meet regularly, fostering empowerment and community engagement.

In early 2024, South Verona CMHS launched the basis to build a recovery college, an educational initiative designed to empower individuals to take control of their own well-being through shared learning and community support [34,72]. Furthermore, the DMH launched another MHRS training program for mental health professionals, reflecting ongoing efforts to integrate recovery principles within clinical practice.

## 4. Discussion

This pilot study aimed to evaluate the feasibility, acceptability, and impact of incorporating a recovery-oriented approach into routine clinical practice at an Italian CMHS. The findings contribute to a broader understanding of CMHS, extending beyond the Italian context and offering insights relevant to global mental health systems.

The study demonstrated that integrating a recovery-oriented approach into routine clinical workflow is feasible, as evidenced by the successful recruitment of mental health professionals (*N* = 19, 66.7%) and service users (*N* = 25). The structured training program, coupled with ongoing supervision and monitoring, ensured consistent application of the MHRS. However, despite these positive indicators, certain challenges emerged, particularly in adhering to intervention timelines, underscoring the necessity for greater flexibility. This finding aligns with the existing literature [10,28], which highlights the tension between structured interventions and the inherently individualized nature of recovery. The focus groups demonstrated the feasibility of implementing recovery-oriented practices despite the challenges of fluctuating attendance, suggesting that adaptive strategies can enhance sustainability.

The acceptability of the MHRS was reflected in positive feedback from both professionals and service users. Professionals noted that MHRS facilitated a shift towards a more recovery-oriented environment, encouraging user involvement in SDM and increasing their motivation for work. These results reinforce previous research [5,23,73,74] advocating for SDM as a core component of recovery-oriented care. Focus groups played an essential role in fostering dialogue and collaboration, promoting an “expert to expert partnership” model. While initial hesitations were reported, over time, participants engaged in meaningful discussions that strengthened their understanding of recovery. The study further highlighted the need for additional training and a shared conceptual framework to facilitate effective collaboration.

A key takeaway is that cultural shifts within CMHS are essential to embedding recovery-oriented practices more deeply into clinical settings [25,26,75].

The study’s impact was evident for both service users and professionals. Service users benefited from improved identification of their needs, leading to a reduction in unmet care gaps [46] and notable improvements in psychosocial functioning and personal recovery. The findings align with evidence suggesting that recovery-oriented tools enhance user engagement and support progress in key recovery domains, including self-care, social relationships, and functional autonomy [55,76,77]. These positive changes, while evident, were primarily observed in users still in the ‘Believing’ phase of the recovery process, underscoring the ongoing nature of recovery. Additionally, the identification of lower levels of change in addiction-related areas, along with subsequent goal setting in this domain, demonstrates the utility of MHRS in detecting emerging challenges and addressing them effectively. The MHRS tool not only captures these shifts but also guides targeted rehabilitation efforts and boosts user motivation.

Professionals also reported increased job satisfaction and engagement, attributed to improved trust between professionals and users and a greater emphasis on SDM. The implementation of the MHRS tool and the emphasis on SDM in focus groups facilitated more empathetic therapeutic relationships, reinforcing the idea that collaboration fosters better outcomes. However, challenges related to intervention adherence suggest that flexibility is crucial in recovery-oriented service delivery, given the unpredictable nature of personal recovery [33,71,75].

This pilot study contributes to the global understanding of CMHS by demonstrating the feasibility of recovery-oriented practices in routine clinical settings, even within traditionally medicalized models of care. The findings have implications beyond Italy, particularly for systems seeking to transition toward recovery-based frameworks. Future research should explore adaptive models that balance structure with flexibility, ensuring that interventions remain person-centered without imposing rigid constraints. The establishment of a self-help group following the pilot study underscores the potential for sustainable, peer-led recovery initiatives, aligning with international evidence on the effectiveness of peer support in enhancing long-term recovery outcomes [78,79,80,81]. Further investigations should assess the longitudinal impact of recovery-oriented approaches, examining their influence on both service users and professionals over extended periods.

### Strengths and Limitations

This small, exploratory study requires caution in interpreting its findings. The pilot involved a limited group of users and professionals from the South Verona CMHS, making it non-representative of the entire service population.

Furthermore, a significant limitation of this study is the absence of a control group. However, given the exploratory nature of the pilot, its primary aim was to assess feasibility and acceptability as a precursor to a larger, controlled study, and the focus was on these aspects rather than achieving a fully representative sample. While the sample may not fully reflect the broader population, it was balanced across key demographics, such as accommodation type (private vs. supported), sex, and diagnoses, in line with the overall clinical data of the Verona DMH [82].

The study’s limitations include potential natural improvements over time (regression to the mean effect) and reduced statistical power due to multiple tests. Further, case-controlled studies are needed to confirm the tool’s effectiveness.

Another limitation is that, although this pilot offered an opportunity to integrate recovery into the service, service users were not involved in its design.

## 5. Conclusions

The recovery-oriented pilot study in the South Verona CMHS demonstrated the feasibility, acceptability, and positive impact of implementing recovery-oriented practices in an Italian CMHS. The service successfully addressed three of Shepherd et al.’s ‘10 key organizational challenges’ for recovery in mental health services [47]: increasing personalization and choice, redefining service user involvement, and transforming the workforce.

Our research findings provide valuable insights for mental health professionals, administrators, and policymakers seeking to enhance mental health services in Italy and beyond. The study underscores the feasibility and acceptability of recovery-oriented practices while emphasizing the need for ongoing efforts to achieve full implementation and integration. Structural changes may take time, but training professionals and users in personal recovery principles can foster a cultural shift and transform service delivery [28].

Notably, the pilot study introduced new interactions based on SDM between users and professionals, while the workforce embraced recovery concepts, leading to a redefinition of user involvement. Sustained efforts are essential to making CMHS more user-centered and responsive to recovery needs and principles (CHIME framework) [71]. Expanding the study to include a larger participant base could further promote the adoption and integration of recovery practices within the service organization. Future initiatives should prioritize the expansion of MHRS training, the development of user facilitators and peer experts, and the inclusion of more participants to solidify recovery-oriented practices and create a more responsive and inclusive mental health service frameworks.

## Figures and Tables

**Table 1:** Summary of MHRS acceptability and feasibility assessment.

Domain	Professionals (*N* = 22)	Service Users (*N* = 21)
**Difficulty of MHRS Implementation**		
Negotiating different visions between user and professional	3.4 (0.7)	3.5 (0.7)
Ensuring equal participation in MHRS	3.3 (0.8)	3.4 (0.8)
Assigning responsibility in care planning	3.2 (0.9)	3.3 (0.8)
Adhering to intervention plan schedule	2.8 (1.0)	3.0 (0.9)
Completing MHRS within two weeks	2.9 (1.1)	—
**Achieved Results**		
Strengthening user involvement in decision-making	4.0 (0.6)	4.1 (0.5)
Identifying new solutions for user needs	4.1 (0.5)	4.0 (0.5)
Uncovering previously hidden resources	4.0 (0.6)	4.1 (0.6)
Improving user-professional trust	3.9 (0.8)	4.0 (0.7)
Increasing user engagement in their care plan	4.2 (0.5)	4.1 (0.7)

Likert scale: 1 = Very False/Very Difficult, 5 = Very True/Very Easy; Mean (SD).

**Table 2:** Sociodemographic and clinical characteristics of service users at BL and FU.

	BL (*N* = 25) Mean (SD)	FU (*N* = 25) Mean (SD)	*p*-Value *
*Male*	15 (60.0%)		
Age (years), mean (SD)	41.0 (9.9)		
*Marital status*			
Single	19 (76.0%)	16 (64.0%)	**<0.001**
Married or in partnership	6 (24.0%)	9 (36.0%)	
*Education*			
Low education	13 (52%)	13 (52%)	-
High education	12 (48%)	12 (48%)	
*Work*			
Employed (Work, Student, Stage, …)	9 (36.0%)	14 (56.0%)	**<0.001**
Unemployed (Housewife, Retired, …)	16 (64.0%)	11 (44.0%)	
*Housing*			
Private accommodation	14 (56.0%)	14 (56.0%)	-
Supported housing	11 (44.0%)	11 (44.0%)	
*Primary clinical diagnosis (DSM 5)*			
Schizophrenia Spectrum Disorder	17 (68.0%)		
Other diagnosis (Mood disorders, Personality disorders, …)	8 (32.0%)		
*Contact with CMHS (years) mean (CI)*	16 (12.5–19.5)		
*Acute ward admission in the previous 6 months*	0.3 (0.5)	0.1 (0.3)	0.059
*Number of psychiatric drugs’ intake*	2.9 (1.6)	2.8 (1.8)	0.746
*Concurrent physical comorbidity*	8 (32.0%)	8 (32.0%)	-
*Current addiction (tobacco, alcohol, cannabinoid, ludopathy)*	0.8 (1.1)	2.8 (1.8)	**<0.001**

* p < 0.05—Bold values indicate statistical significance.

**Table 3:** Recovery Star, functioning, psychopathology, autonomy, needs of care (staff/user perspectives) from BL to FU.

	BL (*N* = 25) Mean (SD)	FU (*N* = 24) Mean (SD)	*p*-Value *
MHRS, Mean (SD)	6.1 (1.5)	6.6 (1.4)	**0.003**
	(*N* = 24 couples)	(*N* = 23 couples)	
1. Managing mental health	5.8 (1.8)	6.6 (1.7)	**0.004**
2. Physical health and self-care	6.7 (2.3)	7.25 (2.1)	0.062
3. Living skills	6.0 (2.0)	6.5 (1.8)	**0.012**
4. Social networks	5.5 (2.2)	5.8 (2.2)	0.142
5. Work	5.5 (2.4)	6.0 (2.4)	**0.007**
	(*N* = 24 couples)	(*N* = 23 couples)	
6. Relationships	5.5 (2.6)	6.2 (2.6)	**0.018**
7. Addictive behavior	7.0 (3.4)	7.0 (3.1)	0.914
8. Responsibilities	8.1 (2.3)	8.4 (1.97)	**0.05**
9. Identity and self-esteem	6.0 (1.8)	6.5 (1.8)	**0.016**
10. Trust and hope	6.9 (1.9)	6.6 (2.0)	0.11
FPS, Mean (SD)	53 (19.7)	62.2 (12.8)	**0.015**
HoNOS Mean (SD)	12.6 (5.5)	9.69 (4.6)	**0.001**
MPR, Mean (SD)	8.7 (1.5)	9.3 (1.5)	**0.003**
CAN *Key-professionals*			
Total mean (SD) needs	10.8 (4.5)	9.6 (5.0)	0.056
Total (SD) met needs	7.7 (3.7)	7.6 (3.8)	0.776
Total (SD) unmet needs	3.0 (0.5)	2.0 (2.1)	0.063
Ratio met/unmet needs	2.6	3.8	
CAN *Service users*			
Total mean (SD) needs	9.8 (4.6)	8.7 (4.6)	0.78
Total (SD) met needs	7.2 (3.94)	7.3 (4.16)	0.94
Total (SD) unmet needs	2.6 (2.78)	1.4 (1.74)	**0.026**
Ratio met/unmet needs	2.8	5.2	

* *p* < 0.05—bold values indicate statistical significance; MHRS—Mental Health Recovery Star. FPS—Personal and Social Functioning Scale; HoNOS—Health of the Nation Outcome Scale; MPR—Monitoring of the Path of Rehabilitation; CAN—Camberwell Assessment of Needs.

**Table 4:** Chosen area/s for the MHRS intervention plan.

	BL (*N* = 25)	FU (*N* = 20)
1. Managing mental health.	5 (20%)	5 (25%)
2. Physical health and self-care.	5 (20%)	5 (25%)
3. Living skills.	4 (16%)	5 (25%)
4. Social networks.	5 (20%)	5 (25%)
5. Work.	12 (48%)	9 (45%)
6. Relationships.	7 (28%)	5 (25%)
7. Addictive behavior.	1 (4%)	4 (20%)
8. Responsibilities.	2 (8%)	2 (10%)
9. Identity and self-esteem.	2 (8%)	3 (15%)
10. Trust and hope.	1 (4%)	0 (0%)

## Data Availability

Requests for original (fully anonymized) participant data may be made to the corresponding author.
